# Microfluidic Passive Flow Regulatory Device with an Integrated Check Valve for Enhanced Flow Control

**DOI:** 10.3390/mi10100653

**Published:** 2019-09-27

**Authors:** Xinjie Zhang, Zhenyu Zhang

**Affiliations:** 1 College of Mechanical and Electrical Engineering, Hohai University, Changzhou 213022, China; 2 School of Mechanical Engineering, and Jiangsu Key Laboratory for Design and Manufacture of Micro-Nano Biomedical Instruments, Southeast University, Nanjing 211189, China

**Keywords:** microfluidic, flow check valve, flow regulating valve, flow autoregulation

## Abstract

A passive microvalve has appealing advantages in cost-effective and miniaturized microfluidic applications. In this work, we present a passive flow regulatory device for enhanced flow control in a microfluidic environment. The device was integrated with two functional elements, including a flow regulating valve and a flow check valve. Importantly, the flow regulating valve could maintain a stable flow rate over a threshold liquid pressure, and the flow check valve enabled effective liquid on/off control, thus accurate forward flow without any backward leakage was achieved. The flow performance of the flow regulating valve was analyzed through 3D FSI (Fluid-Structure Interaction) simulation, and several key parameters (i.e., fluidic channel height and width, control channel length, and Young’s modulus) were found to influence valve flow rate directly. To examine the flow characteristics of the device, we fabricated a prototype using 3D printing and UV laser cutting technologies, and the flow rates of the prototype under varied test pressures were measured in forward and reverse modes, respectively. Experimental results showed that nearly a constant flow rate of 0.42 ± 0.02 mL s^−1^ was achieved in the forward mode at an inlet pressure range of 70 kPa to 130 kPa, and liquid flow was totally stopped in the reverse mode at a maximum pressure of 200 kPa. The proposed microfluidic flow regulatory device could be employed for accurate flow control in low-cost and portable Lab-on-a-Chip (LoC) applications.

## 1. Introduction

Microvalves are vital in various microfluidic applications, e.g. cell separation [1,2,3], droplet manipulation [4,5], liquid mixing [6], and drug delivery [7,8]. Consequently, many microfluidic valves with different structures and functions have been developed for Lab-on-a-Chip (LoC) devices. According to the working principle of a microfluidic valve, the reported valves can be classified as active type and passive type [9]. Active valves usually use external actuators such as pressurized gas [10,11,12], magnetic [13,14], electric [15,16], or thermal forces [17] to adjust the flow resistances of microchannels. As many actuators are highly sensitive, liquid can be controlled consciously and precisely, which enables the high compatibility of active valves in microfluidic large-scale integration [18,19,20]. However, the external actuator also increases the complexity of the valve control system, which is not suitable for the true miniaturization of complex systems.

A passive valve is an alternative solution for effective flow control in a microfluidic system, and it usually relies on a microchannel structure or natural effect to regulate the flow rate. As a complex actuator is not required, a passive valve can be employed for low-cost and portable microfluidic applications. To control liquid in a passive manner, a capillary effect is widely used in many passive valves. As capillary force relies on the cohesion of liquid molecules and adhesive forces between a liquid and microchannel, liquid flow rate can be regulated through adjusting the surface hydrophobicity of the microfluidic structures [21,22]. Despite their simplicity in flow control, the flow rates of such passive valves induced by capillary forces are relatively low and can only be applied for a limited time [23]. In addition, capillary force is usually not stable, which limits its application to some single step biochemical assays. Membrane valve is the other type of passive valve in a microfluidic device, and it is mainly actuated through changing the flow resistance of a microchannel to regulate the flow rate. A membrane valve usually consists of two channels, including a fluidic channel and control channel, which are separated by a thin elastic membrane. When liquid flows into the fluidic channel and the control channel simultaneously, the control channel is pressurized to deflect the membrane, which changes the cross-section of the fluidic channel to regulate the flow rate. A significant advantage of the membrane valve is its flow autoregulatory ability. As flow resistance of the valve is self-adaptive with the varied pressure, a stable flow rate can be achieved. Thus far, a passive membrane valve mainly has two types, which are the single membrane valve and the parallel membrane valve. A single membrane valve usually employs a horizontal membrane [24,25,26] or a vertical flap [27,28] to regulate the flow rate. Similar to push-up or push-down active membrane valves proposed by Quake [29], the horizontal membrane can be deflected up or down the fluidic channel by the liquid pressure of the control channel in the passive membrane valves. Different from the horizontal membrane valve, the vertical flap valve does not have a control channel, and the vertical flap embedded in the fluidic channel is directly deflected by the liquid pressure of the fluidic channel. Similar to a single membrane valve, a parallel membrane valve employs two horizontal [30,31] or two vertical membranes [32] to regulate the flow rate. Since two parallel membranes are deflected to squeeze liquid together, the flow rate is increased to be saturated quickly, and a stable outflow can be achieved at a lower threshold pressure. Although passive membrane valves maintain stable flow rates under varied pressures, they cannot control liquid flow in a backward direction. Since the membranes are deformed under reverse pressures to increase the cross-section of the fluidic channel, continuously increased flow rates are produced in a backward direction [26,27,28,33]. When many passive valves are used in a microfluidic device for complex sample controls through large-scale integration, sample liquids that are output by some valves may flow into the microchannels from the outlets of the other valves to produce a reverse flow due to the unbalanced flow resistance among the valves [2]. The reverse flow may disturb the accurate sample delivery in the microchannels, which finally deteriorates the function of the microfluidic device.

In this work, we developed a passive microfluidic flow regulatory device that could avoid backflow in the process of liquid delivery. The device included a flow regulating valve and a flow check valve. The flow regulating valve was used to regulate the flow rate of liquid, and the flow check valve was used to cut off the reverse flow. Since the flow check valve only allowed forward liquid flow in the microchannels, when liquid flowed into the valve in a backward direction, the valve could block the microchannels and stop the reverse flow immediately. The working principle of the device was demonstrated at first, and the device structure and fabrication process were then described. To investigate the flow characteristics of the device, we solved the 3D FSI (Fluid-Structure Interaction) models of the flow regulating valve and studied the key parameters influencing the flow rate. We also fabricated a prototype device and examined its flow performances in forward and reverse modes through experiments.

## 2. Materials and Methods 

### 2.1. The Working Principle of the Device

**Figure 1:**
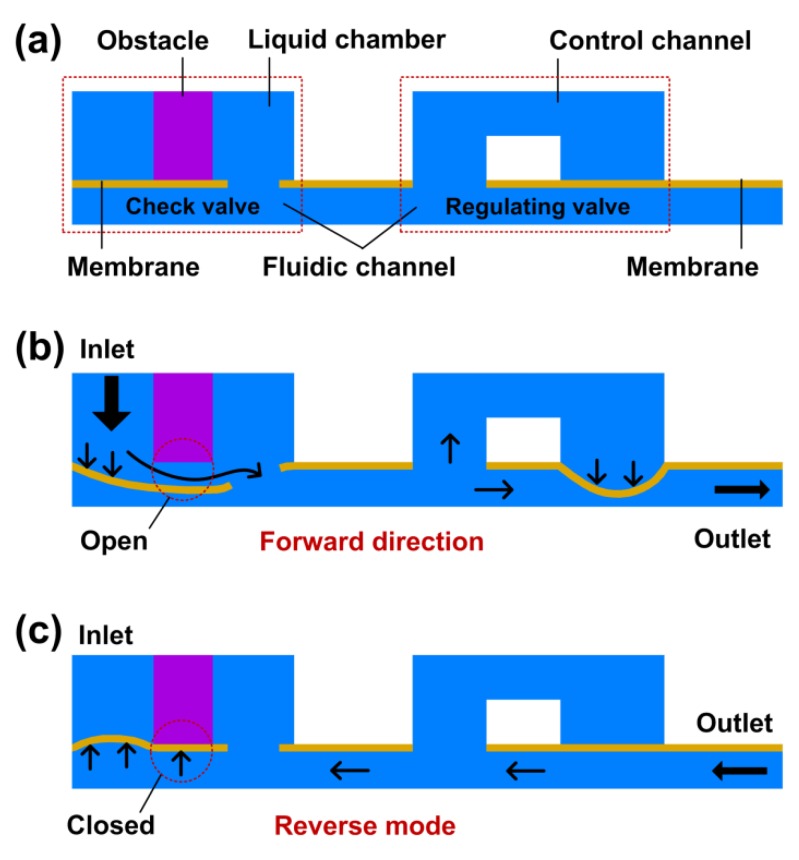
The working principle of the microfluidic flow regulatory device. (**a**) Concept microchannel structure of the device; (**b**) Flow check valve is opened to input the liquid in the forward mode, and a flow regulating valve controls the liquid flowing through the microchannel; (**c**) The flow check valve is closed to stop the liquid in the reverse mode.

The concept design of the microfluidic flow regulatory device is shown in Figure 1a. The device was composed of a flow regulating valve and a flow check valve. The flow check valve included a liquid chamber, an obstacle, a fluidic channel, and an elastic membrane with a hole. The flow regulating valve included a shared fluidic channel, a control channel, and an elastic membrane. When liquid pressure was applied to the device inlet in a forward direction, the membrane of the flow check valve was pushed and deflected towards the fluidic channel (Figure 1b). The flow check valve was then opened to input liquid through a hole, and the liquid flowed into the flow regulating valve directly. When inlet pressure was increased, the elastic membrane of the flow regulatory valve was deflected towards the fluidic channel due to the increased pressure in the control channel. As the deformed membrane reduced the cross-section of the fluidic channel, the flow resistance of the device was increased. When the inlet pressure was higher than a threshold, the increment of flow resistance compensated the pressure increment, thus a constant outflow was achieved in the forward mode. When the device worked in reverse mode, liquid flowed into the fluidic channel from the outlet (Figure 1c). The flow regulating valve did not work due to the balanced liquid pressure between the control channel and the fluidic channel. The liquid then flowed into the flow check valve and pushed the membrane towards the inlet. Since the obstacle was isolated by the inlet and the liquid chamber, the membrane could be tightly compressed to the tip of the obstacle due to the high liquid pressure applied on the membrane contacting the obstacle, thus the flow check valve was closed to stop the liquid in the reverse mode.

### 2.2. Device Design

**Figure 2:**
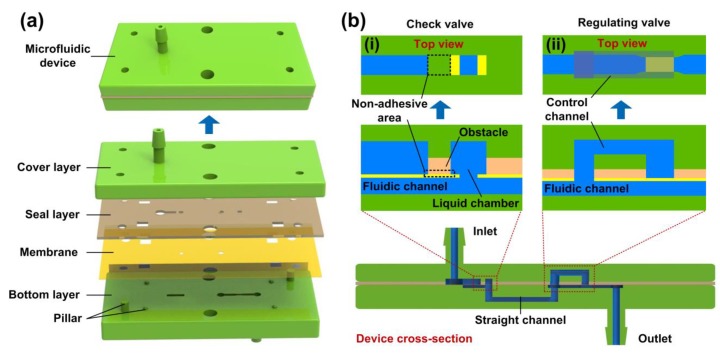
Schematic structure of the microfluidic flow regulatory device. (**a**) Explosive view of the device; (**b**) Microchannel structures of the device. Inset images (i) and (ii) illustrating the top views and the cross-section views of the flow check valve and the flow regulating valve. The black dashed frame indicates the non-adhesive area in the flow check valve.

The schematic structure of the microfluidic flow regulatory device is shown in Figure 2a. The device was composed of four independent parts, which were the cover layer, seal layer, membrane, and bottom layer. Each part was designed using Pro/Engineer software. The cover layer and the bottom layer were designed with microchannels to pass liquid. The seal layer was used for sealing the cover layer and the bottom layer, which prevented liquid leakage between the two layers. The membrane was highly elastic and deformable under liquid pressure. For the sake of liquid infusion, the inlet and outlet tubes were directly designed in the cover layer and the bottom layer, respectively. We also designed some cylindrical pillars and holes in each part for precise assembly. Figure 2b shows the cross-section of the device. Two microvalves were formed in the assembled device, which were the flow check valve (inset image (i)) and the flow regulating valve (inset image (ii)). The inlet of the flow check valve was connected to the device inlet, and the outlet of the flow regulating valve was connected to the device outlet. A straight channel was designed in the bottom layer to connect the two valves. In the flow check valve, an obstacle and a liquid chamber were designed in the seal layer which was attached by a membrane with a square hole, and a fluidic channel was designed in the bottom layer. Since the membrane was not bonded to the obstacle in the black dashed frame, the flow check valve could be opened or closed by pushing the membrane towards the fluidic channel or the inlet hole. In the flow regulating valve, a control channel and a fluidic channel were designed in the cover layer and the bottom layer, respectively. The control channel and the fluidic channel shared a same liquid inlet, and the end of the control channel was separated from the fluidic channel by the membrane. To seal the two valves, the silicon layer and the membrane were irreversibly bonded together, except for the non-adhesive area. 

### 2.3. Device Fabrication

**Figure 3:**
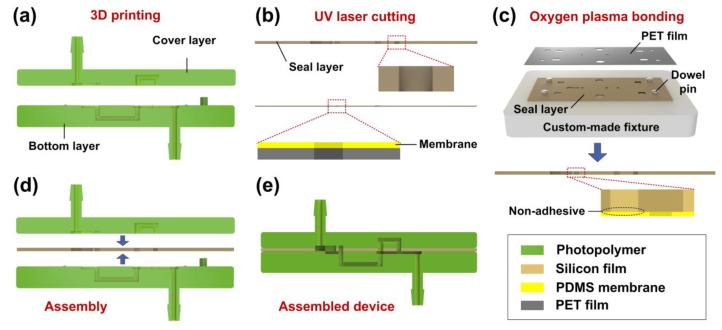
Schematic fabrication process of the microfluidic flow regulatory device. (**a**) 3D printing for the cover layer and the bottom layer of the device; (**b**) UV laser micromachining for the seal layer and the membrane; (**c**) Oxygen plasma treatment for the bonding of the seal layer and the membrane; (**d**) Assembling the device by stacking all the parts layer by layer; (**e**) The assembled device.

The schematic fabrication process of the microfluidic flow regulatory device is shown in Figure 3. First, photopolymer (SOMOS Imagine 8000, DSM, Shanghai, China) was used to fabricate the cover layer and the bottom layer using 3D printing technology (Figure 3a). Then, the seal layer (silicon film) and the membrane (polydimethylsiloxane membrane covered with polyethylene terephthalate film) were micromachined using a UV laser machine (AWAVE 355-10W-30K, Advanced Optowave Corporation, New York, NY, USA), as shown in Figure 3b. Next, a custom-made fixture was employed for precisely assembling the membrane and the seal layer. The fixture was made by transparent acrylic, and four steel dowel pins were installed in the fixture (Figure 3c). To bond the seal layer and the membrane, the two parts were exposed to oxygen plasma at first. Then, the two parts were assembled on the fixture through the dowel pins layer by layer. The membrane should be carefully pressed to the surface of the seal layer to avoid any air bubbles in the interface of the two parts. In the end, the polyethylene terephthalate (PET) film was peeled from the polydimethylsiloxane (PDMS) membrane after the two parts were permanently bonded together, and the bonded parts were used for device assembly. It was noted that the obstacle of the seal layer should not be bonded to the membrane due to the actuation of the flow check valve. The obstacle was then shaded with a slice of paper for avoiding plasma irradiation in the bonding process. After all of the parts were prepared, we sandwiched the bonded seal layer and the membrane between the cover layer and the bottom layer through the cylindrical pillars and the holes (Figure 3d,e). 

### 2.4. Experimental Setup

**Figure 4:**
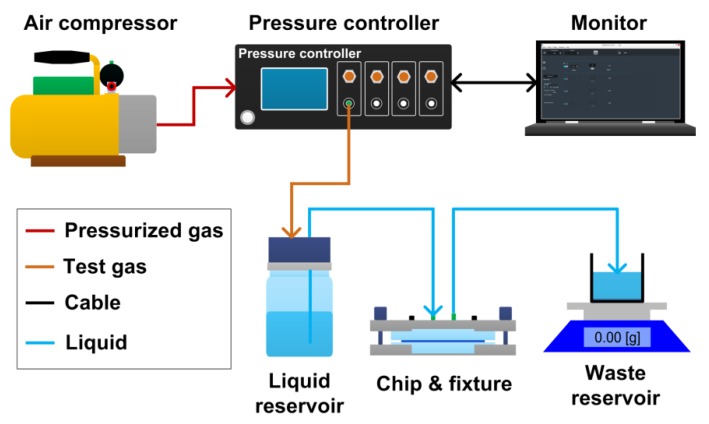
Schematic experimental setup for measuring the flow rate of the microfluidic flow regulatory device.

The experimental setup for the flow rate measurement of the microfluidic flow regulatory device is shown in Figure 4. The setup was composed of an air compressor, a pressure controller (OB1 Base MkIII, Elveflow, Paris, France), a computer monitor, a liquid reservoir, a fixture holding the microfluidic device, a waste reservoir, and an electronic balance (AX523ZH, OHAUS, New Jersey, NJ, USA). In the setup, the air compressor output pressurized air to the pressure controller, which regulated the pressurized air to target the test pressure. The test pressure was then applied to the liquid reservoir to push deionized water flowing into the microfluidic device. Finally, the outflow from the device flowed into the waste reservoir, which was measured by the electronic balance. The flow rate of the microfluidic device was obtained by calculating the water mass change during one minute.

## 3. Results and discussion

### 3.1. Fluid-Structure Interaction (FSI) Modeling

**Figure 5:**
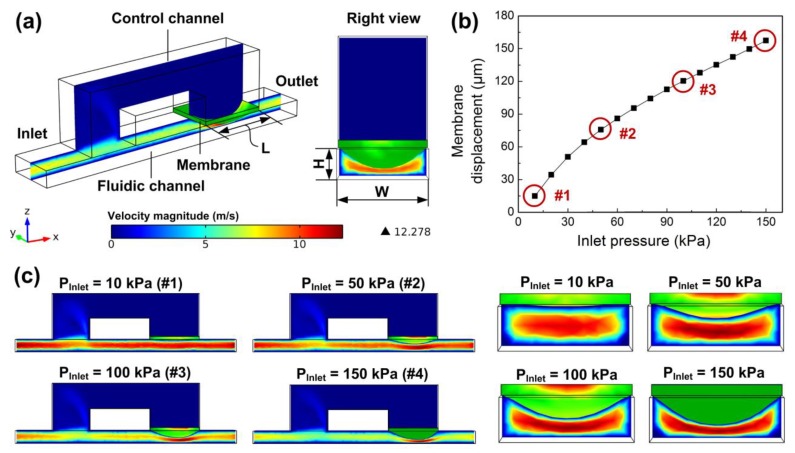
Fluid-Structure Interaction (FSI) model of a flow regulating valve. (**a**) Axonometric and right views of the FSI model, with the color bar representing the velocity magnitude of the liquid domain; (**b**) Membrane displacement versus inlet liquid pressure increasing from 10 kPa to 150 kPa; (**c**) Membrane deformations under different inlet liquid pressures of 10 kPa, 50 kPa, 100 kPa, and 150 kPa.

The forward flow rate of microfluidic flow regulatory device is mainly dependent on the flow rate of the flow regulating valve. To investigate the flow performance of the valve, we built a fully-coupled 3D FSI model of the valve using COMSOL Multiphysics^®^ software (Figure 5a) (Version 4.3b, COMSOL). In the FSI model, both the fluidic channel and the control channel were full of liquids, and a solid membrane was constructed to separate the two channels. An incompressible Navier-Stokes model was used in the liquid domain, and the liquid was set as water with a viscosity of 0.001 Pa s at room temperature. The membrane in the solid domain was set as PDMS with Young’s modulus of 1.7 MPa, which was provided by PDMS membrane supplier, and the Poisson’s ratio of the membrane was set to 0.49 [34]. By applying liquid pressure in the channel inlet and solving the model, the membrane was deflected towards the fluidic channel, influencing the liquid stream in the cross-section (right view in Figure 5a). Figure 5b,c show the membrane deformation under different liquid pressures increasing from 10 kPa to 150 kPa. The fluidic channel height and width were 200 μm and 600 μm, respectively, and the control channel length was 800 μm. It was found that the deflection displacement of the membrane increased towards the fluidic channel when the pressure was increased from 10 kPa to 150 kPa (Figure 5b). The cross-section of the fluidic channel was then decreased with the gradually increased membrane profile, which further squeezed the water flowing through the fluidic channel (Figure 5c), thus the flow resistance of the valve was also increased with the increased inlet pressure. Since the self-adaptive flow resistance continuously compensated the pressure increment, the flow rate of the valve could be regulated automatically.

### 3.2. Numerical Simulation Analysis

**Figure 6:**
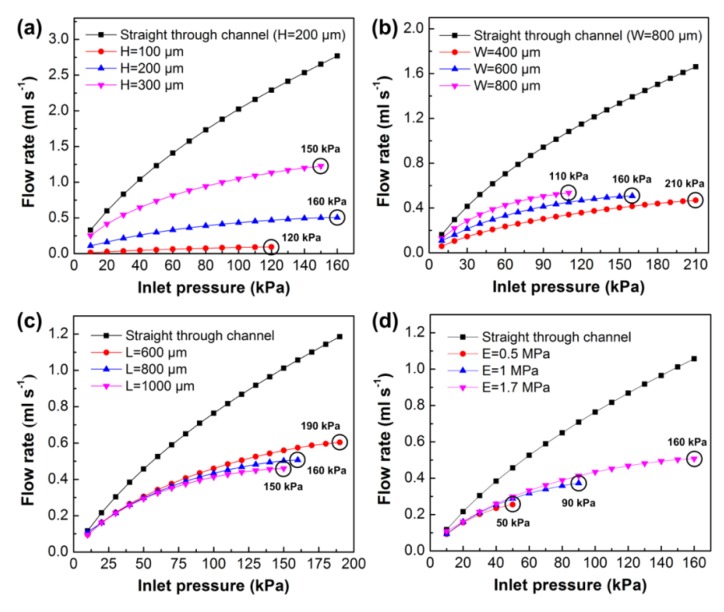
Simulation of the flow rate curve for various parameters of (**a**) fluidic channel height (H), (**b**) fluidic channel width (W), (**c**) control channel length (L), and (**d**) Young’s modulus (E). The circle in the figure indicates the saturation pressure of the model in the simulation process. The saturation pressure represents the maximum pressure of the model before simulation fails.

We next investigated the effects of key parameters (i.e., fluidic channel height and width, control channel length, and Young’s modulus) on the flow rate of the flow regulating valve through numerical simulation. Figure 6a shows the effect of fluidic channel height on the flow rate. The fluidic channel height was set to 100 μm, 200 μm, or 300 μm for three valve models. The other valve parameters were set to the initial values of the basic model in the last section. Each of the models were solved to output maximum flow rate till failure happens due to the membrane contacting the channel wall or membrane fatigue. Simulation results showed that the flow rates of the three models increased at first, and the model with a higher channel height output had a much higher flow rate. The model with a channel height of 100 μm saturated at a pressure of 120 kPa, and the other two models saturated at pressures of 160 kPa (H = 200 μm) and 150 kPa (H = 300 μm), respectively. To analyze the flow regulation abilities of the three models, we chose the last five flow rates of the models and calculated the mean flow rate and the flow variation of each model. The flow variation was defined as the relative pulsation to the mean flow rate, and it was calculated as a ratio of the bilateral tolerance of minimum to maximum flow to the overall mean flow rate. We found that the mean flow rate of the model with a channel height of 200 μm (flow rate of 0.491 ± 0.019 mL s^−1^, flow variation of 3.87%) had the best flow stability compared to the other two models (flow rate of 0.085 ± 0.009 mL s^−1^ and flow variation of 10.59% for model H = 100 μm; flow rate of 1.164 ± 0.066 mL s^−1^ and flow variation of 5.67% for model H = 300 μm). We also calculated a model of a straight through channel without a membrane (channel height of 200 μm), and its mean flow rate was 2.532 ± 0.239 mL s^−1^ with a flow variation of 9.44%. Comparing the above four models, we found that the flow regulating valve had the ability to regulate the flow rate automatically, and the model with the fluidic channel height of 200 μm had the potential to maintain a constant flow rate under varied pressures.

Then, we investigated the effect of fluidic channel width on the flow rate (Figure 6b). The fluidic channel width was set to 400 μm, 600 μm, or 800 μm, and the other parameters were fixed to the initial values of the basic model. The saturation pressures of the three models were 210 kPa (W = 400 μm), 160 kPa (W = 600 μm), and 110 kPa (W = 800 μm), respectively. Although the model with a wider channel achieved a much higher flow rate, it failed at a lower pressure due to a low aspect ratio of the fluidic channel. We also calculated the mean flow rates of the three models and the model with a straight through channel (W = 800 μm). The calculated flow rates were 0.45 ± 0.02 mL s^−1^ (flow variation of 4.44%, W = 400 μm), 0.491 ± 0.019 mL s^−1^ (flow variation of 3.87%, W = 600 μm), 0.5 ± 0.039 mL s^−1^ (flow variation of 7.8%, W = 800 μm), and 1.556 ± 0.106 mL s^−1^ (flow variation of 6.81%, straight channel). We found that the flow variations of the two models with channel widths of 400 μm and 600 μm were much smaller than the other two models, and they maintained stable flow rates at varied pressures. The effect of control channel length on the flow rate is shown in Figure 6c. The control channel length was set to 600 μm, 800 μm, or 1000 μm, and the other parameters were the same as the initial models. Simulation results showed that the longer control channel had a much lower saturation pressure, and the mean flow rates were 0.584 ± 0.022 mL s^−1^ (flow variation of 3.77%, L = 600 μm), 0.491 ± 0.02 mL s^−1^ (flow variation of 4.07%, L = 800 μm), 0.446 ± 0.016 mL s^−1^ (flow variation of 3.59%, L = 1000 μm), and 1.1 ± 0.087 mL s^−1^ (flow variation of 7.91%, straight through channel). Finally, we studied the effect of Young’s modulus of the PDMS membrane on the flow rate (Figure 6d). As the material elasticity of the PDMS membrane was dependent on Young’s modulus, changing Young’s modulus would also change the flow rate of the valve. In the simulation, the Young’s modulus was set to 0.5 MPa, 1 MPa, or 1.7 MPa. As shown in the figure, with the increase of Young’s modulus, the saturation pressure of the model was also increased. Since the membrane with a high Young’s modulus was difficult to be deflected, the model with a higher Young’s modulus also obtained a higher flow rate. It could be concluded from the simulation results that the flow rate of the model was directly proportional to the fluidic channel height, width, and Young’s modulus, while the flow rate was inversely proportional to the control channel length. We found that the model with the fluidic channel height of 200 μm and width of 600 μm had the best flow stability compared to the other models. Although the models with the different control channel lengths had apparently different flow rates, the control channel length had little effect on the flow variation, and all of the models achieved good flow stability.

### 3.3. Experimental Examination

**Figure 7:**
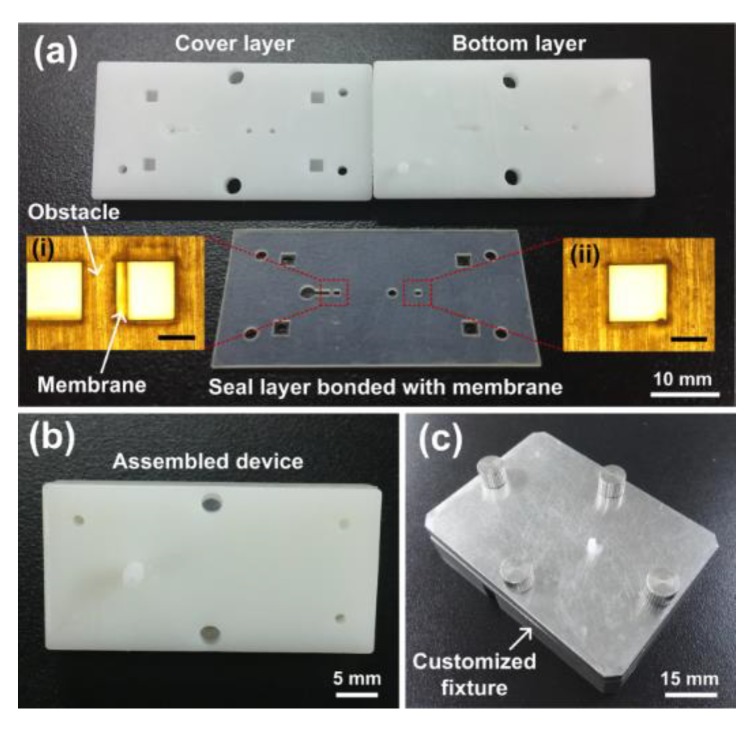
Prototype of the microfluidic flow regulatory device. (**a**) Cover layer, bottom layer, and seal layer with a bonded membrane. Inset images (i) and (ii) illustrating the enlarged membrane structures of the flow check valve and the flow regulating valve. Scale bar 500 μm; (**b**) Assembled prototype; (**c**) Customized fixture holding the prototype.

According to the simulation results, we fabricated a prototype of a microfluidic flow regulatory device using 3D printing and UV laser cutting methods [35]. The prototype parts of the device are shown in Figure 7a. The thickness of the silicon seal layer and the PDMS membrane were 500 μm and 50 μm, respectively. The obstacle in the seal layer was 800 μm in length and 500 μm in width, and the square hole in the membrane was 800 μm in side length (inset image (i)). The fluidic channel height and width were 250 μm and 600 μm, and the control channel length was 800 μm (inset image (ii)), respectively. The straight channel for connecting the two valves was 800 μm in diameter. The assembled prototype is shown in Figure 7b. To examine the flow characteristics of the prototype, a customized steel fixture was fabricated, and the prototype was held in the fixture (Figure 7c). Finally, the prototype was installed in the experimental setup for flow rate measurement.

**Figure 8:**
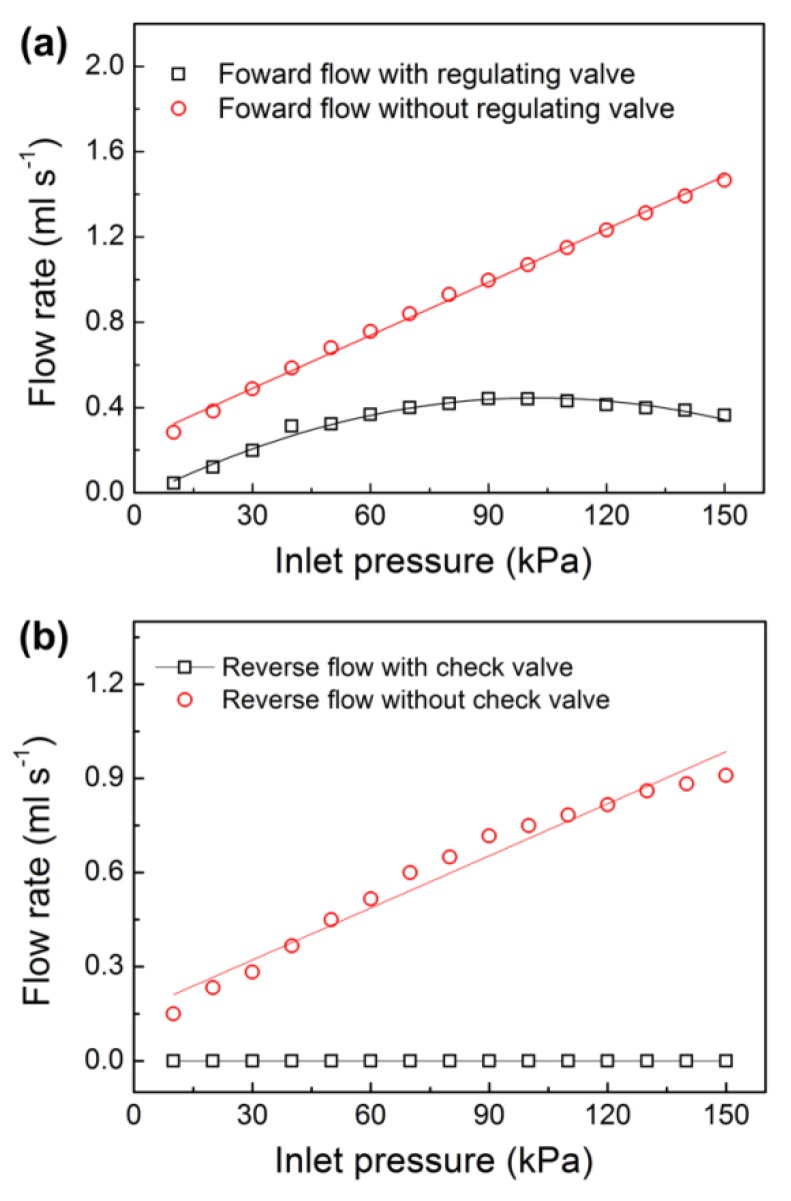
Measured flow rates of the microfluidic flow regulatory device under test pressures from 10 kPa to 150 kPa in (**a**) Forward mode and (**b**) Reverse mode.

The microfluidic flow regulatory device has different flow performances in the forward and reverse modes due to the combination of the flow check valve and the flow regulating valve. To investigate the flow characteristics of the prototype in the forward mode, we first measured the forward flow rate of the prototype. In the experiment, the inlet pressure of the prototype was set to be continuously increased from 10 kPa to 150 kPa, and the flow rates versus the varied pressures are shown in Figure 8a. It was found that the flow rate increased linearly with the increase of the inlet pressure from 10 kPa to 60 kPa at first. When the pressure was higher than 60 kPa, the flow rate increased nonlinearly and achieved a maximum value at a pressure of 90 kPa. Then, the flow rate decreased slowly against the increased pressure. Importantly, the outflow maintained nearly constant under the pressures from 70 kPa to 130 kPa. The calculated mean flow rate under the above pressure range was 0.42 ± 0.02 mL s^−1^, which produced a small flow variation of 4.76%. In the end, the flow rate decreased gradually till the end of the experiment. We did not show the flow rate at pressures higher than 150 kPa because the flow rate continuously decreased and could not be regulated to be constant at much higher pressures. After that, we examined the flow stability of the prototype at pulse pressures of 70 kPa to 130 kPa. The flow rate was measured at every pulse pressure with a test interval of five minutes. We found that the obtained flow rates were nearly identical to those of the records in the continuous experiment. Therefore, we could conclude that the prototype had good responsiveness for flow autoregulation. We also fabricated a prototype without the flow regulating valve for comparison. Since the prototype only had a flow check valve and could not regulate the flow rate by itself, the outflow increased linearly during the whole experimental process. The flow rate of the comparison prototype under inlet pressures from 70 kPa to 130 kPa was 1.076 ± 0.237 mL s^−1^ with a flow variation of 22%. Comparing the two prototypes, we found that the prototype with a flow regulating valve had good flow autoregulation ability and the forward liquid flow was well regulated to be constant under varied pressures.

A check valve (i.e., a one-way valve) can only allow liquid to pass in one direction and it is vital for avoiding backflow in microfluidic systems [36,37]. Due to the unique liquid on/off control function, a microfluidic check valve can be used for effective sample control in low-cost microfluidic devices. For example, varieties of microfluidic check valves have been applied in polymeric micropumps for handy and continuous liquid deliveries [38,39,40]. To examine the liquid cut-off function of the flow check valve in our microfluidic flow regulatory device, we measured the flow rate of the prototype in reverse mode. The liquid pressure was applied in the outlet of the prototype, and the test pressure was still increased from 10 kPa to 150 kPa. The experiments showed that the prototype stopped liquid flow in the reverse mode and no liquid leakage was found in the inlet (Figure 8b). We also examined the long-term durability of the prototype at a much higher pressure of 200 kPa, and we found that the prototype could withstand the high test pressure without any leakage for 24 h, and no burst, leakage, or failure was observed in the prototype. For comparison, another prototype without the flow check valve was fabricated, and it showed the linearly increased back flow in the reverse mode, which was similar to the research findings of the previously reported passive membrane valves. Therefore, we thought the flow check valve in our microfluidic flow regulatory device was effective for avoiding backflow in microchannels. To compare our device with the traditional check valves, the traditional valves were only used for liquid on/off switch operations, and they could not regulate the flow rate in microchannels. However, our device was integrated with the flow regulating valve and the flow check valve together, and it was capable of maintaining constant liquid delivery without any backward leakage. The significant flow characteristics of the device enabled it to be used in many low-cost and portable microfluidic applications. For example, the device could be used as a micropump for continuous and precise sample delivery.

## 4. Conclusions

In summary, we developed a microfluidic flow regulatory device with an integrated flow check valve and flow regulating valve. The device was designed with laminated multilayer structures and was fabricated using 3D printing and UV laser cutting technologies. To obtain stable and controllable outflow through the device, the FSI model for studying flow characteristics of the flow regulating valve was solved, and several key parameters influencing the flow rate of the valve were investigated. Simulation results showed that fluidic channel height and width, control channel length, and Young’s modulus had intimate relationships with the valve flow rate. As the flow rate was increased with the increase of the fluidic channel height and width, while it was decreased with the increase of the control channel length and Young’s modulus of the membrane, the flow rate could be easily changed by varying the above parameters. Based on the theoretical findings in the simulation results, we fabricated a prototype of the microfluidic device, and the flow performance of the prototype device was investigated in our experiment. It was found that the device achieved a stable flow rate at a pressure range of 70 kPa to 130 kPa in forward mode, while zero liquid leakage at a maximum pressure of 200 kPa was realized in reverse mode. Due to the significant flow autoregulation function of the flow regulating valve and the liquid on/off switch operation of the flow check valve, our microfluidic flow regulatory device could be employed for accurate liquid delivery without backflow leakage. The enhanced flow regulation characteristic of the microfluidic flow regulatory device makes it well suited for low-cost and miniaturized microfluidic devices as well as portable in situ monitoring applications.
